# Neuroelectric Mechanisms of Delayed Cerebral Ischemia after Aneurysmal Subarachnoid Hemorrhage

**DOI:** 10.3390/ijms23063102

**Published:** 2022-03-13

**Authors:** Hidenori Suzuki, Fumihiro Kawakita, Reona Asada

**Affiliations:** Department of Neurosurgery, Mie University Graduate School of Medicine, Tsu 514-8507, Mie, Japan; fxmx0216@yahoo.co.jp (F.K.); reona.asd@gmail.com (R.A.)

**Keywords:** cortical spreading depolarization, delayed cerebral ischemia, early brain injury, excitotoxicity, glutamate, inflammation, microcirculation, receptor, seizure, subarachnoid hemorrhage

## Abstract

Delayed cerebral ischemia (DCI) remains a challenging but very important condition, because DCI is preventable and treatable for improving functional outcomes after aneurysmal subarachnoid hemorrhage (SAH). The pathologies underlying DCI are multifactorial. Classical approaches to DCI focus exclusively on preventing and treating the reduction of blood flow supply. However, recently, glutamate-mediated neuroelectric disruptions, such as excitotoxicity, cortical spreading depolarization and seizures, and epileptiform discharges, have been reported to occur in high frequencies in association with DCI development after SAH. Each of the neuroelectric disruptions can trigger the other, which augments metabolic demand. If increased metabolic demand exceeds the impaired blood supply, the mismatch leads to relative ischemia, resulting in DCI. The neuroelectric disruption also induces inverted vasoconstrictive neurovascular coupling in compromised brain tissues after SAH, causing DCI. Although glutamates and the receptors may play central roles in the development of excitotoxicity, cortical spreading ischemia and epileptic activity-related events, more studies are needed to clarify the pathophysiology and to develop novel therapeutic strategies for preventing or treating neuroelectric disruption-related DCI after SAH. This article reviews the recent advancement in research on neuroelectric disruption after SAH.

## 1. Introduction

A rupture of an intracranial aneurysm causes subarachnoid hemorrhage (SAH), for which the prognosis remains poor [1]. Aneurysmal rupture-induced elevation of intracranial pressure (ICP) and extravasated intracranial blood components trigger early brain injury (EBI) and systemic complications such as cardiopulmonary dysfunction and systemic inflammatory response syndrome, which are sometimes fatal [2,3]. Delayed cerebral ischemia (DCI) is an important modifiable prognostic factor and develops at day four or later post-SAH in patients surviving the initial aneurysmal rupture [4]. Cerebral vasospasm has classically been considered to be only cause of DCI, but now multiple concurrent and synergistic mechanisms have been suggested as a cause of DCI [5]. EBI may be a precursor or a contributor to DCI, and therefore some pathophysiologies may be shared or interrelated between EBI and DCI [6]. These shared or interrelated pathophysiologies may include cortical spreading depolarization (CSD) [7], which is intimately related to epileptic discharge and excitotoxicity, leading to metabolic derangement, that is, mismatch of metabolic supply and demand, with the resultant relative cerebral ischemia and neuronal death [8,9]. In fact, recent clinical studies reported that CSD and epileptic discharge were in frequently observed in association with the development of DCI after aneurysmal SAH [10,11]. A common inducer of CSD and epileptic discharge, glutamate, was also reported to increase in brain parenchyma after SAH, followed by the development of DCI [12]. In this review, the authors focus on a potential mechanism of glutamate-mediated neuroelectric disruption in the development of DCI after aneurysmal SAH.

## 2. Glutamate

Brain tissues contain high concentrations of free glutamates, in the 5–15 mmol/kg range intracellularly, which are involved in endogenous neural signaling in multiple pathways as a major excitatory amino acid neurotransmitter throughout the central nervous system [13]. Glutamates are released from the presynaptic vesicles, while excess glutamates are removed from the extracellular space by astrocytes and endothelial cells with uptake and metabolizing functions, and are exhausted into the blood through diffusion when endothelial glutamate concentrations become higher than in the blood [14]. Thus, pathologically excessive glutamates are caused by a derangement between glutamate release and reuptake.

### 2.1. Signaling via Glutamates

In normal brain, glutamate is the most abundant neurotransmitter used by excitatory synapses [15]. Once released into the synaptic cleft from presynaptic vesicles in neurons and astrocytes, glutamates activate postsynaptic glutamate receptors, which consist of ionotropic glutamate receptors and metabotropic glutamate receptors (mGluRs). Ionotropic glutamate receptors are ligand-gated ion channels regulating fast synaptic transmission, including α-amino-3-hydroxy-5-methyl-4-isoxazolepropionic acid (AMPA; GluA1–4), N-methyl-D-aspartate (NMDA; GluN1, GluN2A–D, and GluN3A, B), kainate (GluK1–5), and δ (GluD1 and GluD2) receptors, and mGluRs (mGluR1–8) G-protein coupled receptors, leading to activation of intracellular metabolic pathways to modulate postsynaptic responses, synapse activities, and glutamate releases and to regulate different cell functions ranging from cell cycle to gene expression [16,17,18,19]. AMPA receptors are the first ones to be activated by released glutamates and then to depolarize the postsynaptic membrane, defining the strength of the postsynaptic responses to activate downstream signaling pathways, resulting in the propagation of the excitatory signal [15]. That is, synaptic activity initially stimulates the influx of sodium ions (Na^+^) through AMPA receptor channels, which increases the concentrations of positively charged ions in the cytoplasm, causing cell depolarization [20]. As a result, NMDA receptor channels become permeable to calcium ions (Ca^2+^), and Ca^2+^ influx activates Ca^2+^/calmodulin-dependent protein kinase II, protein kinase C, protein kinase A, and tyrosine kinase, which phosphorylate AMPA and NMDA receptors [20]. The affinity of AMPA receptors for glutamates is relatively low, and the number of glutamate molecules bound to AMPA receptors determines the open probability; in contrast, NMDA receptors have a higher affinity for glutamates and desensitize slower than AMPA receptors, but the slow binding rate puts a considerable limit on the opening probability of NMDA receptors during the short-lived glutamate peak [21]. This neurotransmitter system not only drives physiological excitatory signal transmission as well as abnormal hyperexcitable circuitry during a seizure but also may initiate neurodegenerative processes by excessive calcium uptake and pushing cells toward apoptotic cell death [22]. 

### 2.2. Major Glutamate Receptors

AMPA receptors are highly dynamic receptors formed by tetrameric assembly of four subunits, GluA1–4, and their functional properties largely depend on the composition of these subunits [23]. AMPA receptors are primarily of the GluA1/GluA2 and GluA2/GluA3 configuration [17]. GluA1–4 are widely expressed in both neurons and glia, but the predominantly expressed subunits are GluA1 and GluA2 [24]. GluA1–3 are expressed in the majority of neurons in the nervous system of mature animals, while GluA4 is primarily expressed early in development and in cerebellar granule neurons and some populations of interneurons in the mature brain [25]. The protein levels of GluA1 subunits were higher than that of GluA2 subunits in mature neurons [26]. AMPA receptors are not normally Ca^2+^ permeable, by virtue of their GluA2 subunits [27]. AMPA receptors lacking GluA2 subunits or containing unedited (Q form) GluA2 subunits are rendered permeable to Ca^2+^ [17]. The subunit switch of GluA1 and GluA2 may lead to the excessive intracellular Ca^2+^ influx, resulting in neuronal injury after SAH [28]. 

NMDA receptors are composed of three subunits (GluN1–3) and are involved in various processes from learning and memory to neurodegeneration [19]. At the synaptic level, GluN2A is activated to mediate the prosurvival signaling, while abruptly elevated extracellular glutamates stimulate the extrasynaptic GluN2B to trigger excitotoxic neuronal death [18]. As well, under nonexcitotoxic conditions, mGluR1α couples to the neuroprotective phosphoinositide 3-kinase (PI3K)-Akt signaling cascades [29]; however, under excitotoxicity, excess stimulation of NMDA receptors activates Ca^2+^-dependent protease calpain, which causes calpain-mediated truncation of mGluR1α at Ser^936^ [30]. The truncated mGluR1α disrupts the link between the PI3K-Akt signaling and mGluR1α, while the truncated mGluR1α-mediated intracellular Ca^2+^ release from the endoplasmic reticulum is maintained and contributes to Ca^2+^ overloading through the enhancement of NMDA receptor-mediated Ca^2+^ influx [30]. The truncated mGluR1α translocates to axons and enhances glutamate releases and thereby excitotoxicity, suggesting that there is a positive feedback loop between the truncation of mGluR1α and excitotoxicity [30]. Endogenous releases of glutamates and NMDA receptors were also reported to be involved in both CSDs and epileptiform activities [31].

There are eight major mGluRs and several splice variants, subclassified into three groups (I, II, and III) based on the structure, G-protein coupling or function, and ligand selectivity: Group I (mGluR1 and mGluR5) is linked to activation of phospholipase C (PLC); and Groups II (mGluR2 and mGluR3) and III (mGluR4, mGluR6, mGluR7, and mGluR8) are linked to inhibition of adenylate cyclase to change levels of cyclic adenosine monophosphate [32,33]. mGluR1 is one of the most abundantly expressed mGluRs in the mammalian brain [16]. mGluR modulates synaptic transmission and plasticity and probably plays no primary roles in mediating excitotoxic brain injuries; however, mGluR influences excitotoxic injuries and can be the secondary therapeutic target in strokes [34]. Group I mGluRs-activated PLC generates diacylglycerol and inositol 1,4,5-trisphosphate triggering Ca^2+^ release from endoplasmic reticulum, which together through protein kinase C can enhance excitotoxic Ca^2+^ entry through NMDA receptors, reverse operation of the electrogenic Na^+^/Ca^2+^ exchangers and membrane Na^+^/hydrogen ions (H^+^) exchangers, and activate phospholipase A2, promoting reactive oxygen species (ROS) formation and lipid peroxidation [13]. In contrast, Group II and III mGluRs usually exert inhibitory effects on neural circuits and thereby anti-excitotoxic effects [35], although neuronal mGluR2 activation may enhance excitotoxicity, possibly by limiting the release of γ-aminobutyric acid (GABA) [36]. 

### 2.3. Glutamate in Blood Vessels

AMPA, NMDA, kainate receptors, and mGluRs are all expressed on cerebral microvascular cells and perivascular astrocytic processes [37,38,39]. Glutamates contribute to the dilatory tone of cerebral microvessels under physiological conditions via these receptors [40]. In normal brain tissues with preserved neurovascular coupling, an increase in neuronal activities is associated with arteriolar dilation to increase local blood flow (functional hyperemia) via glutamate release [5]. That is, the activated neuron releases glutamates, which bind to at least mGluRs and NMDA receptors on perivascular astrocytes and trigger intracellular Ca^2+^ increases to activate large-conductance Ca^2+^-activated potassium ions (K^+^) channels for outflow of K^+^ at astrocytic end-feet [5,40]. As a result, overall mild increases in perivascular K^+^ concentrations (<20 mM) hyperpolarize arteriolar smooth muscle cells and induce vasorelaxation [41]. Glutamates have been also demonstrated to increase vascular permeability through activation of NMDA receptors in rat cerebral cortex [42] and induce an increase in cultured brain endothelial cell permeability via the action on endothelial AMPA, NMDA, kainate receptors, and group I and III mGluRs [43,44]. Glutamate-mediated activation of group I or III mGluRs disturbs the barrier function of endothelial cells by promoting dephosphorylation of vasodilator-stimulated phosphoproteins (VASPs), which increases actin filament formation as well as cell retraction, thus impairing cell-cell junctions [43,45]. On binding of glutamates, mGluR1 and mGluR5 activate multiple intracellular signaling pathways, such as PLC, protein kinase C, and mitogen-activated protein kinase pathways [16]. mGluR1 and mGluR5 expressed on glial and Purkinje cells are activated by glutamates and are reported to promote the secretion of thromboxane A2, endothelin-1, and 20-hydroxyeicosatetraenoic acid by these cells, leading to constriction of microvessels [46,47]. 

### 2.4. Glutamate in SAH

**Figure 1:**
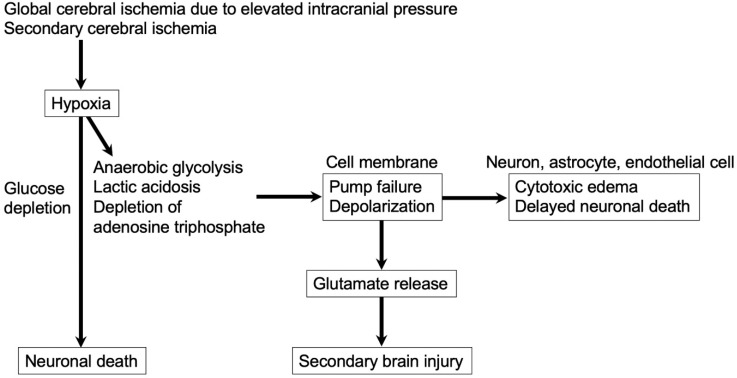
Glutamate release at the rupture of intracranial aneurysm. Released glutamate causes secondary brain injury.

Aneurysmal rupture-induced elevated ICP and the subsequent global cerebral ischemia induce energy storage loss, metabolic failure, and disturbed ionic hemostasis, resulting in plasma membrane depolarization, which causes an excessive and uncontrolled release of neurotransmitters such as glutamates (Figure 1) [48,49]. Neurons release glutamates under decreased cerebral blood flow of less than 20 mL/100 g brain/min [50]. SAH increases the permeability of the paravascular space, through which blood components and the degradation products are perfused into the brain parenchyma, inducing intraparenchymal microvascular constriction, inflammation, and microthrombus formation [51]. Post-SAH blood-brain barrier (BBB) disruption is also associated with abluminal and intraparenchymal platelet aggregates [41]. Although massive SAH and secondary tissue ischemia induce ROS and proinflammatory cytokines, which damage arterial, capillary and venous endothelial cells to activate inflammatory cells and platelets, leading to microthrombus formation throughout the entire cerebral hemisphere even distant from the aneurysm rupture site [5], platelet-mediated microthrombi were reported to release glutamates [52]. Furthermore, excessive glutamates were synthesized and released by activated astrocytes, microglia, and neutrophils in experimental SAH [4]. On the other hand, excitatory amino acid transporters on astrocytes, which uptake glutamates, were downregulated in experimental SAH, at least partly explaining the excessive glutamates and thereby the excitotoxicity in EBI [53].

In a clinical setting, cerebral glutamate levels are considered to increase within minutes after aneurysmal SAH [12]. Studies using cerebral microdialysis showed that glutamate levels in an acute phase were already high in poor-grade SAH patients with neurological impairments and cerebral edema; a trend toward normalization of the values was associated with clinical improvement, whereas further deterioration led to permanent neurological deficits [12]. In addition, elevated intraparenchymal concentrations of glutamates at 1 to 7 days post-SAH were an independent predictor of DCI and 12-month poor outcomes in clinical settings [12]. Cerebrospinal fluid levels of glutamates were significantly correlated with angiographic vasospasm and DCI [54,55]. In a rat model of SAH, an increase in glutamate concentrations was accompanied by vasospasm of the basilar artery [56].

Excessive glutamate overactivates the receptors, which mediate intracellular Ca^2+^ overload by Ca^2+^ influx from the extracellular space through ionotropic glutamate receptors as well as intracellular Ca^2+^ release from the endoplasmic reticulum via mGluRs [5,57]. Then, excitotoxicity or CSD follows and causes mitochondrial dysfunction and compromised energy metabolism, inducing ionic imbalance; as a result, neurons, astrocytes, pericytes, and vascular endothelial cells constituting the neurovascular unit develop apoptotic or necrotic cell death [5,49]. Persistent CSDs are known to be followed by spreading depression of electrocorticographic activities and increased glutamate releases, the latter of which leads to excitotoxicity [5]. A positive feedback loop was also reported between the stimulation of glutamate receptors such as the NMDA receptor or mGluR1 and glutamate releases [57].

### 2.5. Glutamate and Inverse Neurovascular Coupling after SAH

**Figure 2:**
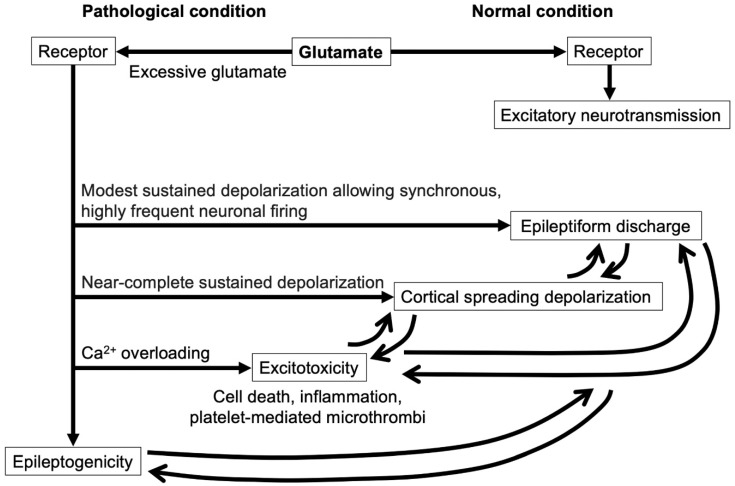
Glutamate action via the receptors. In normal conditions, glutamate is a major excitatory neurotransmitter via the receptor. In pathological conditions, excessive glutamates activate the receptors excessively and cause epileptiform discharges, cortical spreading depolarization, and excitotoxicity, depending on the extent of the cell membrane depolarization. Each of the neuroelectric disruptions triggers the other. Surviving cells irrespective of these events achieve epileptogenicity.

CSD and seizure or epilepsy are distinct entities, but both develop related to glutamate releases after aneurysmal SAH and have similar toxic effects, such as increased metabolic demand, inverse vasoconstrictive neurovascular coupling, disruption of the BBB, and cellular death [4]. CSD may lower the threshold of seizure (inappropriate neuronal firing) [58], while seizure may induce CSD (Figure 2) [59].

In SAH, hemolysis causes an increase in basal perivascular K^+^ concentrations and a decrease in basal nitric oxide (NO) [5]. In addition, infiltration of blood degradation products into intraparenchymal perivascular space induces higher amplitude in spontaneous Ca^2+^ oscillations in hypertrophic astrocyte end-feet surrounding parenchymal arterioles, causing a surge of extracellular and perivascular K^+^ [5]. Thus, a physiological mechanism of neurovascular coupling, that is, activated neuron-induced glutamate release, causes an excessive concentration (>20 mM) and impaired clearance of perivascular K^+^, which result in depolarization of parenchymal arteriolar smooth muscle cells, inducing vasoconstriction or pathological inversion of neurovascular coupling [41]. The phenomenon may form the basis of spreading ischemia and DCI development associated with CSDs after aneurysmal SAH [5]. In experimental SAH models in rats or mice, impaired neurovascular coupling developed time-dependently up to 96 h, and any neuronal or metabolic activation such as sensory stimulation, an increase in carbon dioxide, and a decrease in pH resulted in parenchymal arteriolar constriction, mismatch of cerebral metabolism and blood flow, and relative cerebral ischemia, causing further brain damage after SAH [60].

## 3. Excitotoxity in Post-SAH Ischemic Brain

Excitotoxity contributes to both EBI and DCI after aneurysmal SAH. In EBI, massive aneurysmal rupture causes severe elevation of ICP, followed by transient cerebral circulation arrest, which leads to cessation of neuronal electrical activity within seconds, mitochondrial dysfunction associated with decreased production of adenosine triphosphate to deteriorate the energy state and to disrupt the Na^+^-K^+^ pump, and ion homeostasis, resulting in disturbed membrane ion gradients (depolarization), Ca^2+^ influx, and extracellular release of a large amount of glutamates from depolarized nerve terminals and astrocytes within minutes [61]. In DCI, secondary cerebral ischemia also triggers excessive glutamate releases. Massive releases of glutamates over-activate AMPA, NMDA, and kainate receptors on neurons, as well as other cellular components of the neurovascular unit, causing excessive intracellular Ca^2+^ entry as the primary mediator of excitotoxicity through the receptors, which is augmented by Ca^2+^ releases from endoplasmic reticulum via activation of mGluRs [13,61]. Intracellular Ca^2+^ overload induces further release of glutamates and overactivation of multiple Ca^2+^-dependent enzymes such as calpains, other proteases, protein kinases, calcineurins, endonucleases, phospholipases A_2_, and xanthine oxidases [13]. The activation of theses enzymes impairs mitochondrial energy production and causes increased production of ROS to promote lipid peroxidation, membrane failure, and cell damage, as well as alterations in the organization of the cytoskeleton, activation of genetic signals leading to cell death, and an increase in expressions of immediate early genes [13,33,62]. Cellular damage also causes further glutamate releases [13]. In addition, Ca^2+^ influx activates neuronal NO synthases to produce NO, which may be involved in both normal neuronal signaling and free-radical-mediated glutamate excitotoxicity harnessed by macrophages [13,63]. Ca^2+^ overload and oxidative stress, including NO, cause apoptosis and necrosis, which can occur caspase-dependently or -independently [13]. Glutamates also increase deoxyribonucleic acid binding of the redox-regulated transcription factors, nuclear factor-κB, and activating protein 1, as well as upregulate the immediate early gene, c-fos, leading to glutamate-induced apoptosis or necrosis [33]. Glutamate levels in cerebrospinal fluid and peripheral blood have been reported to be positively correlated with infarct size, infarct growth, and functional outcomes in clinical settings [64]. In contrast, the neuroprotective neurotransmitter GABA rapidly and transiently increases in the extracellular space like most neurotransmitters, but the expressions of both GABA-A and GABA-B receptors decrease after cerebral ischemia to cause impaired GABA-mediated neurotransmission, which contributes to ongoing neuronal excitability and possibly to neuronal death [61]. Excitotoxic neuronal death is not a uniform event but, rather, a continuum of necrotic, apoptotic, and autophagic morphologies [33].

Excitotoxicity is composed of two components: the first one is an acute, intracellular influx of Na^+^ and chloride ions followed by water influx, resulting in cell swelling, tissue edema and, consequently, impaired perfusion of the surrounding brain tissues, even in the absence of extracellular Ca^2+^; the second one is Ca^2+^-dependent delayed cellular degeneration [62]. In contrast to neuronal swelling, which developed immediately after excessive glutamate exposure, delayed neuronal death was observed 24 h post-glutamate exposure and was abolished by the removal of Ca^2+^ while potentiated by the addition of Ca^2+^ [18]. The removal of Na^+^ from the culture medium prior to glutamate exposure prevented neuronal swelling but had no effects on delayed neuronal death [18].

### 3.1. Glutamate Receptors and Ions in Excitotoxicity

Although the NMDA receptor was initially considered to be a critical mediator in focal cerebral ischemia, subsequent studies support a more central role for AMPA receptors in hippocampal injuries associated with global cerebral ischemia [65]. AMPA receptors mediate Na^+^ influx and therefore can contribute to excitotoxic Ca^2+^ overload and neuronal death [13]. Although AMPA receptors lack direct linkage to NO synthases and nicotinamide adenine dinucleotide phosphate (NADPH) oxidases, AMPA receptors participate substantially in brain damage after focal and global ischemia; in global ischemia, AMPA receptors typically contribute more than NMDA receptors to delayed death of selectively vulnerable neurons, possibly due to upregulation of Ca^2+^-permeable AMPA receptors [13]. Ischemia upregulates Ca^2+^-permeable AMPA receptors in vulnerable neuronal populations, which constitute a dominant route for toxic Ca^2+^/zinc ion (Zn^2+^) entry [66].

In addition to Na^+^ and Ca^2+^, H^+^ participates in excitotoxicity. In ischemic brain tissues, extracellular pH typically drops within minutes toward 6.5 or lower due to anaerobic glycolysis for resynthesis of adenosine triphosphate, and an increase in extracellular H^+^ attenuates NMDA receptor channel openings and NADPH oxidase 2 activity, resulting in reduced NMDA receptor-mediated excitotoxicity [67]. However, ischemic acidosis is itself cytotoxic to both neurons and glia by enhancing neurotoxic Ca^2+^ overload via the gating of acid-sensing ion channels and by being accompanied by an increase in intracellular Zn^2+^ [13]. Relatively low concentrations (≈20 μM) of Zn^2+^ elicit apoptotic neuronal death, while higher concentrations (50–100 μM) of Zn^2+^ cause neuronal death, with the characteristics of necrosis [68]. In addition to promoting neuronal death, intracellular release of excitotoxic Zn^2+^ contributes to the death of adjacent non-neuronal cells such as astrocytes, oligodendroglia, and capillary endothelial cells in ischemic brain tissues [13]. An intracellular increase in Zn^2+^ is considered to mediate peroxynitrite-induced death through activation of extracellular signal-regulated kinases 1/2 and arachidonate 12-lipoxygenases and thereby further ROS generation [69], and to upregulate intercellular adhesion molecule-1 expression in vascular endothelial cells, promoting leukocyte attraction and microvascular leakage [70,71]. Several other membrane channels may be activated in part as a result of overstimulation of glutamate receptors and can contribute to toxic Ca^2+^/Zn^2+^ overload and other ionic derangements in ischemic brain tissues [13].

Although excitotoxicity was originally described as specific to neurons, oligodendrocytes and astrocytes also suffer excitotoxic injury and death [13]. Astrocytes are less insensitive to excitotoxicity compared with oligodendrocytes: this is because most astrocytes express AMPA receptors and mGluRs, but NMDA receptors and Ca^2+^-permeable AMPA receptors are generally not abundant [72,73,74]. However, as astrocytes are vulnerable to Zn^2+^ or H^+^-induced damages, astrocytic death may increase secondary to excitotoxicity occurring in nearby neurons or oligodendrocytes [13].

### 3.2. Relationships among Inflammation, Microthrombus, and Excitotoxity

Excitotoxicity per se triggers and augments inflammatory reactions to continue destroying brain tissues. The neurovascular unit-constituent cells release cytokines and chemokines, recruiting leukocytes to the evolving ischemic region over hours to days, and advance microvascular damage and oxidative stress [75,76]. Inducible NO synthases are induced in infiltrating neutrophils and endothelial cells in ischemic brain tissues to produce NO and to synergize oxidatively with superoxide emanating from neutrophil NADPH oxidase 2 and endothelial NADPH oxidase 4, aggravating brain damage [13]. Activated microglia are a significant source of redundant extracellular glutamates that induce excitotoxic neuronal death [33]. Although microglia-mediated neuroinflammation in EBI may have impacts on neuronal excitotoxicity, BBB disruption, and the further changes of immune responses, all of which may lower the seizure threshold, activated microglia themselves may promote epilepsy development independent of the inflammatory responses [77].

In DCI after SAH, platelet aggregates are also observed to be associated with or without focal microvascular constriction and to be extravasated into the brain parenchyma by platelet-mediated release of collagenase and subsequent depletion of collagen IV in vessel walls [78]. The extravasated platelet aggregates or platelet-mediated microthrombi are reported not only to propagate pro-inflammatory signaling [79] but also to release glutamates, causing excitotoxic brain injuries [52]. Although glutamate does not cross the BBB, BBB disruption at sites of microthrombi or extravasated platelets that release glutamates during their lysis or aggregation may allow neurons to be exposed to excessive glutamates [52]. Platelets have dense granules, carrying a considerable amount of glutamates, and also express glutamate receptors on their surface [80]. Excessive glutamate is reported to induce platelet activation and synthesis of thrombogenic peptides, plasminogen activator inhibitor-1, and hypoxia-inducible factor-2α from pre-existing messenger ribonucleic acids in anucleate platelets, which are mediated mostly through AMPA receptors [80].

## 4. CSD

CSDs are slowly self-expanding recurrent waves of intense neuronal and glial mass-depolarization (elevation of postsynaptic membrane potential) and spread in all directions from a region of onset [5]. CSD may be induced by a decrease in oxygen and glucose due to global cerebral ischemia at aneurysmal rupture or subsequent focal cerebral ischemia, as well as a decrease in NO, an increase in K^+^, oxyhemoglobin, endothelin-1, and glutamates in the subarachnoid space after aneurysmal SAH [4,81,82]. CSD is reported to occur spontaneously, with high incidence immediately up to a couple of weeks, peaking at days 5−7, after aneurysmal SAH [83,84]. CSDs are known to cause poor outcomes by increasing metabolic demand, decreasing blood supply, predisposing to seizure activity, and worsening brain edema after SAH [10].

### 4.1. Pathophysiology of CSD

CSDs usually originate from the boundary hypoperfused zone between injured (presumably already depolarized) and relatively normal tissues [85]. CSDs are triggered when the resting-state oxygen supply-demand mismatch in already critically hypoperfused and hypoxic tissues or metastable tissues is transiently worsened by either reduced supply or increased demand, which includes transient systemic hypoxia or hypotension, and functional activation by somatosensory stimuli [85]. As a result, the larger the volume of metastable tissue, the higher the chance of CSD occurrence [10].

Once a CSD originates, it propagates throughout the surrounding tissues and often into healthy brain tissues, although the gyri, sulci, and pial vessels can be barriers to propagation; this is one reason that interpreting surface recordings obtained by subdural electrodes is difficult in the human brain [10]. During CSDs, neuronal membrane potentials approach zero due to opening of non-selective cation channels and transmembrane ion fluxes, resulting in dramatic elevations of extracellular concentrations of K^+^ and H^+^ and intracellular concentrations of Na^+^ and Ca^2+^ [10]. CSDs also disrupt the BBB, resulting in exposure to serum concentrations of ions to decrease extracellular concentrations of magnesium ions and to increase extracellular K^+^ concentrations [86]. The changes in the local microenvironment induce glutamate releases in neurons, contributing to the loss of the normal membrane potential and thereby CSD initiation and excitotoxicity through the receptors [59,86]. Elevated extracellular K^+^ as well as glutamates diffuse into the adjacent brain tissues and trigger the same depolarization cycles, allowing CSDs to propagate at a slow pace of 2–6 mm/min on the gray matter with high neuronal and synaptic density, with a predilection for surrounding injured tissues [10,86,87]. As near-complete depolarization precludes action or postsynaptic potentials, CSDs are thus associated with depression of all spontaneous or evoked cortical electrophysiological activities, that is, they are followed by spreading depression [10]. If mechanisms such as the Na^+^-K^+^ pumps do not work to restore membrane potentials, the affected neurons and astrocytic end-feet swell from an osmotic imbalance with the influx of cations, leading to distortion of neuronal dendritic architecture and spreading depression of electrocorticographic activity [5,86]. When CSD waves recur several times in a short time span, CSDs impose tremendous metabolic burden and paradoxical vasoconstriction, leading to DCI or secondary brain injuries [88]. Persistent CSDs also increase a release of neurotransmitters such as glutamates, causing glutamate-induced excessive stimulation and excitotoxicity [5].

### 4.2. Mechanisms for CSD to Induce DCI or Brain Injuries

**Figure 3:**
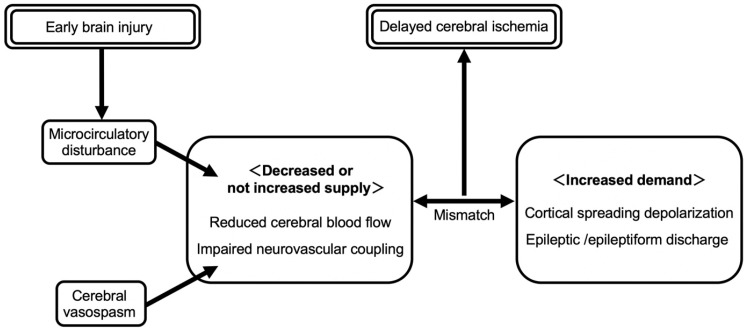
Metabolic supply-demand mismatch hypothesis for delayed cerebral ischemia. When metabolic demand is increased due to depolarizing events such as cortical spreading depolarizations or epileptiform activities and exceeds the impaired blood supply (decreased blood supply or not sufficiently increased blood supply) due to large-vessel vasospasm or microcirculatory disturbance after subarachnoid hemorrhage, a mismatch in neuronal metabolism occurs, resulting in relative cerebral ischemia, that is, delayed cerebral ischemia.

**Figure 4:**
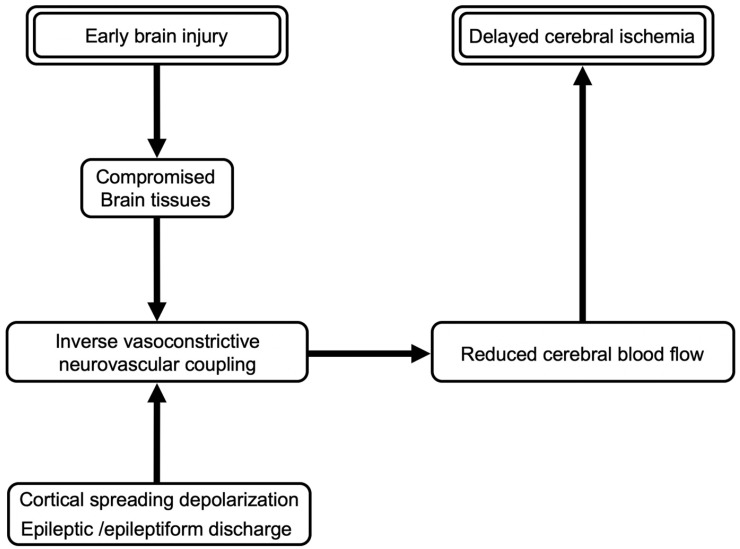
A mechanism for cortical spreading depolarization or epileptiform discharges to cause delayed cerebral ischemia. In the compromised brain after subarachnoid hemorrhage, the neuroelectric disruption increases metabolic demand but decreases cerebral blood flow through inverse vasoconstrictive neurovascular coupling, leading to DCI.

CSD exacerbates brain injuries through the following possible mechanisms. First, CSDs are energetically highly costly and cause heavy metabolic demand, even more than epileptic activities [10]. After CSDs, repolarization to restore normal transmembrane ion gradients requires adenosine triphosphate and phosphocreatine, associated with intense metabolic activation and with increased consumption of oxygen and glucose; therefore, the brain can fully recover from the metabolic challenge owing to a compensatory vasodilatory response (normal neurovascular coupling) via the release of nitric oxide (NO) coupled to CSD under physiological conditions [89]. However, if the resultant hyperemia and improved oxygen delivery do not meet the demand of energy expenditure, CSDs are prolonged, with increasingly harmful consequences, up to neuronal death associated with relative cerebral ischemia and aggravating brain injuries (Figure 3) [86]. Second, CSDs may exert strong vasoconstrictive effects on ischemic or post-SAH brain tissues [90], which further worsen the supply-demand mismatch and are highly detrimental to already metabolically compromised brain tissues [84,85]. This may be because CSD further elevates extracellular K^+^ concentrations that are already high in damaged tissues or due to hemolysis post-SAH, promoting arteriolar vasoconstriction [86]. CSD clusters also reduce NO by oxygen deficiency-induced impaired production as well as oxyhemoglobin’s action, contributing to spreading ischemia and the resultant cortical necrosis on post-SAH brain tissues [49]. In addition, an acidic microenvironment in damaged tissues and persistent CSD-induced decreases in extracellular pH are vasoconstrictive [5,86]. Thus, recurrent CSDs or CSD clusters emerging spontaneously in metabolically compromised tissues after SAH switch the vasodilatory response to an inverse, vasoconstrictive neurovascular coupling, resulting in cortical spreading ischemia; if the vulnerable tissue fails to recover from the depolarization, it leads to irreversible injuries or focal cerebral infarction (Figure 4) [84]. Even if cerebral infarction is not developed, the affected areas are further susceptible to the ongoing spread of CSDs, falling into a vicious cycle [86]. Furthermore, adenosine, a potential vasodilator and a metabolite of adenosine triphosphate degradation, is reduced in metabolically disturbed tissues and may cause the failure to recover vasodilatory reactions after CSDs [91]. In a clinical setting of SAH, a CSD was associated with a transient decrease (indicating increased consumption and reduced delivery of oxygen) and the subsequent transient increase (due to the hyperemic effect) of tissue oxygen pressure in the human cerebral cortex; the former hypoxic phase increased, but the latter hyperoxic phase decreased, correlated with increased frequency of CSDs, and monophasic hypoxic responses to CSD clusters were found predominantly in patients with DCI [84]. Consistent with the findings, increased frequency of spontaneous CSDs was associated with the development of delayed cerebral infarction [92]. Lastly, CSDs are reported to activate inflammatory reactions [93], disrupt the BBB [94], and be prone to epileptic discharges [8], all of which may cause and aggravate brain injuries. In addition to CSDs, seizures and some kind of epileptiform discharges are considered to be involved in inflammatory reactions and the development of cerebral vasospasm, microthrombi, and DCI in severely injured SAH brain [95,96]. Toll-like receptor 4 may be a common signaling pathway in CSD-induced neuroinflammation related to microglia, astrocytes, and neurons, leading to neuronal damage [58].

## 5. Interplay between CSD and Seizure or Epilepsy

CSD is a pathologic disruption of cortical electrical activities (large changes in the slow electrical potentials and silencing of brain electrical activities), resulting from and resulting in spreading the disturbance of ion homeostasis between intra- and extracellular space, leading to neuronal swelling, distortion of dendritic spines, glial depolarization, and damages as well as changes in the local vascular responses [86]. Epileptic activities are characterized by paroxysmal cellular depolarization shifts, which are correlated with a synchronous network event, resulting from possibly synchronous activation of recurrent excitatory paths and the consequent giant excitatory postsynaptic potentials [8]. Because the sustained depolarization underlying ictal epileptic field potentials remains below the inactivation threshold for the action potential-generating channels, neurons can continue synchronous, highly frequent firing that is superimposed on the moderate sustained depolarization [8]. Interictal spikes and ictal epileptic field potentials spread between neurons at a rate usually higher than that of CSD through different mechanisms of spread [97]. Both CSDs with neuronal deactivation (near-complete sustained depolarization of neurons causing spreading depression) and seizures with neuronal activation (modest sustained depolarization allowing synchronous, highly frequent neuronal firing) have excitotoxic states as a common mechanism, and the link between them has been reported after aneurysmal SAH (Figure 2) [8]. Early CSD occurrence may be a risk factor for late post-SAH seizures [8].

CSD may lead to the development of epileptiform activities through the following several mechanisms. First, cortical depression is followed by a neuronal state of hyperexcitability with less hyperpolarization, which may prime damaged neurons for seizure or epileptiform activities [98]. Second, post-CSD cellular swelling may increase epileptiform activities [86]. Third, CSDs may upregulate ionotropic glutamate receptors, which may lower the threshold for epileptiform activities [99]. Endogenous releases of glutamates and the receptors may play central roles in both CSD and epileptiform activities, because antiepileptic NMDA receptor antagonists halted CSDs [31]. Lastly, low concentrations of extracellular magnesium ions and high concentrations of extracellular K^+^ observed in CSDs are known to induce seizure-like activities [100,101]. In addition, impairments of GABA-mediated inhibitions are considered to increase excitability and susceptibility of neuronal tissues to both CSDs and ictal epileptic field potentials and to promote continued seizure activities, although the binding of GABA to postsynaptic receptors by nature causes a decrease in postsynaptic membrane potentials (hyperpolarization) [8]. As BBB disruption is a causative factor for epileptogenicity and is induced by CSDs, CSDs may also contribute to epileptogenicity via BBB disruption after aneurysmal SAH [8].

In contrast, depolarized neurons in CSDs may interrupt the wave of seizures [86]. In addition, it is reported that the depression of electrical activities accompanying CSDs may be the cause of the postictal state [86]. Seizures have been observed before, during, and after CSDs [86].

## 6. Epileptogenicity

Neurons that survive in the penumbra are the underlying substrates for ischemia-induced epileptogenicity [102]. Glutamates have been shown to play roles in the initiation and spread of seizure activities and ischemia-induced epileptogenicity [64]. Surviving neurons with a less severe glutamate insult are considered to induce prolonged, reversible depolarization and therefore epileptogenicity [102]. 

Glutamate receptor activation has been strongly associated with epileptogenicity [102], but none of the currently available antiepileptic drugs has been found to be clinically effective against epileptogenicity [103]. However, an AMPA receptor antagonist, but not a NMDA receptor antagonist, suppressed hippocampal neuronal cell loss and gliosis or epileptogenicity after pilocarpine-induced status epilepticus in rats [104]. Neuronal damage, gliosis, and mossy fiber sprouting have been implicated in epileptogenicity [105]. Astrocytes become reactive after brain injuries and play an important part in the development of secondary brain injuries and epileptogenicity through releasing numerous proinflammatory cytokines [104]. Seizures upregulate GluA2-lacking AMPA receptors, which promote Ca^2+^ entry and cause cell death [106]. NMDA receptor antagonists failed to block the early stage of status epilepticus [107]. AMPA receptors can influence epileptogenicity and seizure generation in different ways, and AMPA receptor modifications may be early mediators of an epileptogenic cascade; in particular, changes in AMPA receptor phosphorylation seem to be important for epileptogenicity [103]. Thus, AMPA receptors have become a therapeutic target due to the involvement in ictogenesis and epileptogenicity [108].

## 7. Glutamate and the Receptors in Animal Models of SAH

Under SAH pathology by endovascular perforation in rats, glutamates in the cerebrospinal fluid were increased and caused BBB disruption and neuronal apoptosis within 72 h [57,109,110]. An intracerebroventricular injection of glutamate aggravated BBB disruption, while inhibition of mGluR1 attenuated BBB disruption and cerebral edema associated with suppression of glutamate-induced VASP downregulation and inactivation as well as aquaporin-4 upregulation [109]. Inhibition of GluN1/GluN2B and mGluR1 decreased glutamate releases and attenuated EBI in terms of neuronal apoptosis through reducing glutamate-induced intracellular Ca^2+^ overload [57]. Post-SAH glutamate-induced excitotoxicity caused calpain-mediated C-terminal truncation of mGluR1α, which suppressed PI3K-Akt signaling and induced caspase-dependent neuronal apoptosis [110]. 

### 7.1. Effects of mGluR Inhibition in Animal Models of SAH

A group I mGluR (mGluR1 and mGluR5) antagonist S-4-carboxyphenylglycine inhibited cerebral vasospasm in the basilar artery in a mouse SAH model by an injection of autologous arterial blood into the cisterna magna [111]. Both mGluR1 and mGluR5 are expressed on endothelial cells and affect endothelial cell function, likely through changes in endothelial cell cytoskeleton [111]. It was also demonstrated that inhibition of mGluR1 prevented delayed vasospasm of the basilar artery by attenuating the downregulation and the inactivation of endothelial NO synthases and VASPs, microthrombus formation in the cerebral cortex, and neuronal death in the CA1 region, with the suppression of mitochondrial-dependent apoptosis pathways at day 7 post-SAH [112].

In filament-perforation SAH rats, mGluR5 was expressed on activated microglia [113]. Pharmacological activation of mGluR5 attenuated microglial activation, cytokine production, brain edema, and caspase-dependent neuronal apoptosis in the cerebral cortex and improved neurological function in EBI at 24 h after SAH [113]. However, it is important to note that activation of mGluR1 and mGluR5 may either amplify or reduce neuronal death, depending on the context and the nature of the toxic insults, while mGluR1 and mGluR5 antagonists are consistently protective in both in vitro and in vivo models of neuronal death [114]. Inhibition of mGluR5 may have neuroprotective effects by suppressing NMDA receptor signaling as well as the mGluR5 pathways [114].

### 7.2. Effects of NMDA Receptor Inhibition in Animal Models of SAH

At 3–5 h after filament-perforation SAH in rats, hippocampal NMDA-receptor subunit mRNA was decreased, possibly providing a neuroprotective mechanism against neuronal death following SAH with moderate hypoperfusion [115]. In the same SAH models, ifenprodil, which is a non-competitive antagonist of GluN1-GluN2B, prevented EBI in terms of SAH-induced neuronal death in the basal cortex and hippocampal CA1 area, BBB disruption, and cerebral edema at 24 or 72 h, and improved functional outcomes at 14 days post-SAH by attenuating glutamate-induced excitotoxicity (cellular and mitochondrial Ca^2+^ overload) [116]. 

In filament-perforation SAH mice, memory impairments were observed at least from 2 to 12 weeks and peaked at 8 weeks post-SAH, associated with interstitial glutamate accumulation in the hippocampus by impaired glutamate uptake due to decreased glutamate transporter (GLT)-1 expression on the membrane of astrocytes [117]. The GLT-1 downregulation transcriptionally occurred and was induced by post-SAH increased histone deacetylase (HDAC) 2 followed by the deacetylation of histones in astrocytes; the resultant long-term accumulation of glutamates in the synaptic space resulted in reduced phosphorylation levels of excitatory glutamate receptors (GluN2B and GluA1) on the postsynaptic membrane and the long-term inhibition of synaptic excitability in the hippocampus [117]. A selective HDAC2 inhibitor prevented post-SAH GLT-1 downregulation, preserved phosphorylation levels of GluN2B and GluA1, and improved neurobehavioral results [117]. In normal conditions, glutamates in the synaptic cleft are rapidly absorbed into astrocytes to maintain the excitability of synapses [117].

### 7.3. Effects of AMPA Receptor Inhibition in Animal Models of SAH

A study reported that a selective AMPA receptor antagonist GYKI-52466 decreased BBB permeability at 3 h after SAH by a prechiasmatic blood injection in rats, but other detailed analyses were not performed [118]. GYKI-52466, a selective and potent AMPA/kainate receptor antagonist, inhibited vasospasm in a rat femoral artery vasospasm model [119]. GYKI-52466 has muscle relaxant and anti-convulsant properties [119]. Filament-perforation SAH caused BBB disruption, brain edema, hippocampal neuronal death, and neurological impairments at 24 h post-SAH, associated with upregulation of phosphatase and tensin homolog deleted on chromosome ten (PTEN) and alterations of AMPA receptor subunits (unchanged expression of GluA1 and decreased expression of GluA2 and GluA3 at cytomembrane) in rats [120]. A PTEN inhibitor attenuated EBI, possibly by modulating AMPA receptor subunits at the cytomembrane (decreased expression of GluA1 and preserved expression of GluA2 and GluA3) at 24 h post-SAH [120]. At 24 h after SAH by a blood injection into the cisterna magna in mice, GluA1 was decreased in the hippocampus [121]. However, it remains unexamined whether or not there is an acute increase in GluA1 within hours of SAH. AMPA receptor trafficking can be modulated by proinflammatory cytokines, with tumor necrosis factor-α rapidly increasing synaptic GluA1 within minutes [122]. At 24 h post-SAH in a rat model of SAH by a blood injection into the prechiasmatic cistern, knockdown of ring finger protein 216, which inhibits degradation of Toll-like receptors, reduced post-SAH increases in cellular apoptosis and microglia and attenuated brain edema and neurological impairments via the prevention of post-SAH upregulation of GluA1 and GluA2 and the decrease of the GluA1/GluA2 ratio by increasing Arc, which is a protein coded by the immediate early gene and closely related to glutamate neurotransmission [28]. An AMPA receptor agonist re-increased brain edema and re-aggravated neurological function [28]. At 24 h after filament-perforation SAH in mice, a selective noncompetitive AMPA receptor antagonist perampanel significantly suppressed post-SAH neurological impairments, brain edema, BBB disruption, epileptiform discharges without obvious convulsion by inhibiting post-SAH activation of GluA1 and GluA2, as well as upregulation of an inflammatory mediator tenascin-C in neurons and capillary endothelial cells [123].

At 6 days post-SAH by a blood injection into the prechiasmatic cistern in rats, platelet-mediated glutamate releases at sites of microthrombosis led to a loss of GluA2 expression on neurons [52]. Exposure of neurons to modestly increased glutamates markedly downregulated surface AMPA glutamate receptors via endocytic machinery [124]. At 6 days pots-SAH in the same model, synapses in neurons in the CA1 area were decreased in the absence of ischemia and neuronal death, accompanied by a reduction in GluA1 and an increase in GluA2 [125].

## 8. Neuroelectric Disruption in a Clinical Setting of Aneurysmal SAH

Seizures with or without convulsions are a well-known predictor of poor outcomes after aneurysmal SAH, but there is no evidence indicating that antiepileptic drugs can improve post-SAH outcomes [126,127]. Although electroencephalography monitoring is in general performed for 24–48 h when used solely for detecting electrographic seizures, some studies have reported that about 10-day continuous electroencephalography monitoring that starts within 48 h of admission is useful for early diagnosis of DCI development [96]. Continuous electroencephalography monitoring has shown a decrease in normal higher frequency activities, with increases in epileptiform abnormalities (sporadic epileptiform discharges, lateralized rhythmic delta activities, lateralized periodic discharges, generalized periodic discharges, or seizures) or pathological low frequency activities (worsening focal slowing) hours to days before the development of DCI [96,128,129]. Ictal-interictal continuum abnormalities, which include sporadic epileptiform discharges (spikes and sharp waves), periodic epileptiform discharges, and rhythmic patterns, share some features with seizures [130]. A large percentage of SAH patients exhibit at least one form of ictal-interictal continuum abnormalities, which develops mostly within the first 3 days of SAH onset and halts by day 10 post-SAH [96]. The development of late-onset (post-SAH day 6 or later) ictal-interictal continuum abnormalities might contribute to the development of DCI, possibly by triggering CSDs, or to a lesser extent, directly [96]. A prospective study reported that predictive values for DCI development were the highest in late-onset epileptiform abnormalities, followed by worsening slowing, possibly being a surrogate downstream from CSDs, on continuous electroencephalography monitoring, and that daily transcranial Doppler ultrasound velocities interrogating vascular supply alone were less predictable [129]. A higher burden of epileptiform abnormalities was also reported to be an independent predictor of 3-month poor outcomes after aneurysmal SAH [11].

To diagnose CSD, intracranial electrocorticography is needed, although ictal-interictal continuum abnormalities may represent a surface electroencephalography surrogate downstream from CSDs [96]. Intracranial electrocorticography suggested that CSDs are the phenomena underlying DCI [131] and demonstrated that CSDs and ictal epileptic field potentials develop simultaneously in a SAH patient [8]. When metabolic demand is increased due to depolarizing events such as CSDs or epileptiform activities and exceeds the impaired blood supply due to large-vessel vasospasm or microcirculatory disturbance after SAH [5], a mismatch in neuronal metabolism occurs, causing progression of CSDs or epileptiform activities, recovery failure from CSDs, and ultimately neuronal death [129].

## 9. Influence of Systemic or Local Inflammation and Glymphatic Impairment on Neuroelectric Disruption-Related DCI

DCI occurs via a complex interplay among several concurrent processes, including cerebral vasospasm (vasospasm in the main trunk of cerebral artery), especially near a ruptured aneurysm, small distal artery vasospasm, microcirculatory disturbance (microvasospasm, microthrombosis, disturbance of venous outflow, compression of vasculature by tissue edema, and disruption of neurovascular coupling), mismatch of metabolic supply and demand, and inflammatory reactions [5]. SAH allows blood components, including oxyhemoglobin, to penetrate into brain parenchyma via paravascular pathways of cerebrospinal fluid, and the impairments of glymphatic systems and meningeal lymphatic drainages lead to blood component stasis in the paravascular spaces, being predisposed to CSDs and seizures [5]. Seizures and CSDs are capable of triggering each other, possibly via facilitating synchrony [132], and seizures and, to an even greater extent, CSDs increase oxygen use within activated cortex and may cause inadequately matched hypermetabolism, worsening post-SAH ischemic brain injuries [10,90]. Tonic-clonic activities at SAH onset aggravate systemic inflammatory response syndrome, which in turn may induce seizures and cause DCI [133,134]. Inflammation increases BBB permeability, which has been linked to seizures, while seizures induce cerebral inflammation, suggesting bidirectional relationships or a vicious cycle between inflammation and seizures [134]. Seizures may also cause cerebral vasospasm in a similar manner as CSDs, with metabolic derangements and localized electrolyte imbalance [135]. As inflammation is an important pathological factor of cerebral vasospasm, microcirculatory dysfunction and DCI after SAH [136,137], as well as ischemic stroke and atherosclerosis [138,139,140], neuroelectric disruption may be involved in a variety of pathologies via interplay with inflammation, driving interest in further research.

## 10. Perspective

Although post-SAH DCI is caused by multiple pathologies, CSDs and epileptic activity-related events may be important contributors to DCI development and ultimately lead to neuronal death through excitotoxicity or the development of cerebral infarction. In addition, each of excitotoxicity, CSDs, and epileptic activities can trigger the other. CSDs, seizures, and periodic discharges are associated with increased metabolic demand and can lead to metabolic crisis and excitotoxicity, especially in situations with reduced brain perfusion, which can occur by many causes, including EBI, brain edema, cerebral vasospasm, and microcirculatory disturbance after SAH. One approach to suppress the development of the neuroelectric disturbances is to control systemic physiological parameters well. This includes the avoidance of hypoglycemia, hypoxia, and hypotension, all of which can trigger CSDs in the compromised brain tissues [10]. Pharmacologically suppressing epileptiform abnormalities or augmenting cerebral perfusion and oxygen delivery also may be useful for preventing DCI, but investigation is needed for whether some kind of antiepileptic drugs or other interventions prevent not only seizures but also DCI. Considering that glutamates and the receptors play central roles in the neuroelectric disruption, the pharmacology targeting them should be a reasonable translational approach for improving functional outcomes after SAH. Many studies have focused on different strategies, including the inhibition of glutamate synthesis, acceleration of glutamate reuptake, blocking of glutamate releases, and antagonization of glutamate actions on the receptors [141]. The approach may be promising, because it has been reported that many antiepileptic drugs and glutamate receptor antagonists can antagonize CSDs [142], and that an AMPA and NMDA receptor antagonist exerted neuroprotective effects on cerebral ischemic lesions in a randomized controlled clinical trial [143]. As seizures or CSDs are merely symptoms, preventive antiepileptic treatments may be justified, but treatments for the underlying cause (i.e., inflammation) should be developed. Currently, the mechanisms of neuroinflammation after SAH are being elucidated, and Toll-like receptor 4 may be a good molecular target [144]. We hope that basic and clinical research on neuroelectric disruption will be advanced to improve outcomes of aneurysmal SAH patients.

## Abbreviations

AMPA, α-amino-3-hydroxy-5-methyl-4-isoxazolepropionic acid; BBB, blood-brain barrier; Ca^2+^, calcium ions; CSD, cortical spreading depolarization; DCI, delayed cerebral ischemia; EBI, early brain injury; GABA, γ-aminobutyric acid; GLT, glutamate transporter; H^+^, hydrogen ions; HDAC, histone deacetylase; ICP, intracranial pressure; K^+^, potassium ions; mGluR, metabotropic glutamate receptor; Na^+^, sodium ions; NADPH, nicotinamide adenine dinucleotide phosphate; NMDA, N-methyl-D-aspartate; NO, nitric oxide; PI3K, phosphoinositide 3-kinase; PLC, phospholipase C; PTEN, phosphatase and tensin homolog deleted on chromosome ten; ROS, reactive oxygen species; SAH, subarachnoid hemorrhage; VASP, vasodilator-stimulated phosphoprotein; Zn^2+^, zinc ions.
